# ClusTRace, a bioinformatic pipeline for analyzing clusters in virus phylogenies

**DOI:** 10.1186/s12859-022-04709-8

**Published:** 2022-05-28

**Authors:** Ilya Plyusnin, Phuoc Thien Truong Nguyen, Tarja Sironen, Olli Vapalahti, Teemu Smura, Ravi Kant

**Affiliations:** 1 Department of Veterinary Bioscience, University of Helsinki, 00014 Helsinki, Finland; 2 Department of Virology, University of Helsinki, 00014 Helsinki, Finland; 3 Department of Virology and Immunology, Helsinki University Hospital, Diagnostic Center, 00029 Helsinki, Finland

**Keywords:** Phylogenetic analysis, Cluster analysis, Variant calling, Virus, SARS-CoV-2

## Abstract

**Background:**

SARS-CoV-2 is the highly transmissible etiologic agent of coronavirus disease 2019 (COVID-19) and has become a global scientific and public health challenge since December 2019. Several new variants of SARS-CoV-2 have emerged globally raising concern about prevention and treatment of COVID-19. Early detection and in-depth analysis of the emerging variants allowing pre-emptive alert and mitigation efforts are thus of paramount importance.

**Results:**

Here we present ClusTRace, a novel bioinformatic pipeline for a fast and scalable analysis of sequence clusters or clades in large viral phylogenies. ClusTRace offers several high-level functionalities including lineage assignment, outlier filtering, aligning, phylogenetic tree reconstruction, cluster extraction, variant calling, visualization and reporting. ClusTRace was developed as an aid for COVID-19 transmission chain tracing in Finland with the main emphasis on fast screening of phylogenies for markers of super-spreading events and other features of concern, such as high rates of cluster growth and/or accumulation of novel mutations.

**Conclusions:**

ClusTRace provides an effective interface that can significantly cut down learning and operating costs related to complex bioinformatic analysis of large viral sequence sets and phylogenies. All code is freely available from https://bitbucket.org/plyusnin/clustrace/

**Supplementary Information:**

The online version contains supplementary material available at 10.1186/s12859-022-04709-8.

## Background

Emerging pathogens are a constant threat to mankind, as illustrated by the West Africa Ebola [1] and Zika [2] virus outbreaks in 2014 and 2015, respectively, and the ongoing Severe Acute Respiratory Syndrome Coronavirus 2 (SARS-CoV-2) pandemic. These viruses are of zoonotic origin, like the majority of emerging pathogens [3–5]. Wild animals host a vast reservoir of pathogens and these can spill over to human populations under adequate conditions [4, 5]. Anthropogenic disturbances in high biodiversity regions, new forms of land use, increasing human and production animal densities, climate change, travel and globalization have dramatically increased this risk [4, 6]. The impact on human healthcare and economics has been illustrated by SARS-CoV-2 pandemic that has caused numerous deaths and human suffering, delivery and workforce shortages, travelling limitations, and many other disturbances to both business and normal life activities [4].

All virus genomes change over time due to mutations introduced in the viral genome, primarily by errors made by viral polymerases during replication [7]. However, most changes have minor effect on the phenotype of viruses. However, some mutations may affect the key pathogenic properties of the virus, such as transmissibility and disease severity, or the performance of vaccines, therapeutic agents or diagnostic tools [7].

The rapid progress in sequencing technologies has provided an opportunity to study viral molecular epidemiology and evolution in nearly real-time [8]. The current COVID-19 is the first pandemic with the pathogen being under surveillance using full genome sequencing on a global scale and over an extensive time period [9]. Surveillance of the pandemic creates demand for fast and scalable sequencing, genome assembling, viral strain assignment, phylogenetic analysis, variant calling and molecular epidemiology to inform contact tracing and non-pharmaceutical interventions. Although bioinformatics offers an abundance of methods and tools for sequence analysis, their employment in virology and epidemiology can be hindered by the developer-user gap between bioinformatics and other fields [10]. This gap can be bridged by pipelines tailored specifically for the analysis of viral sequences and equipped with intuitive interface and output reporting.

SARS-CoV-2 is the causative agent of coronavirus disease 2019 (COVID-19) [11]. The SARS-CoV-2 pandemic has already infected more than 437 million people in 224 countries, causing nearly 6 million deaths globally as of 1st of March 2022 (https://www.worldometers.info/coronavirus/).

SARS-CoV-2 is a global challenge, which is further complicated by the continuous emergence of new Variants of Concern (VOCs) or Variants of Interest (VOI). Variants that have carried VOC status include Alpha (B.1.1.7) [12], Beta (B1.351) [13], Gamma (P.1) [14], Delta (B.1.617.2) [15] and, as of writing this, we are experiencing the spread of Omicron variant (B.1.1.529) [16]. These VOCs pose an increased public health risk due to having one or more of the following characteristics: higher transmissibility [17], immune escape properties for antibodies from previous infection [18], lower response towards current vaccines compared to the original wild type strains these vaccines were based on [19]. Detecting and monitoring these novel variants is essential in SARS-CoV-2 surveillance.

A number of bioinformatic software packages are already available to help with detection, tracking and tracing of SARS-CoV-2 variation e.g. Pangolin [20], Nextstrain [21], Nextclade [22], Jovian [23], HaVoC [24] and Lazypipe [25]. Such tools are certainly helping the global effort for COVID-19 surveillance, but they are not void of limitations. Tools like Pangolin and Nextclade are primarily designed for tracking large accumulations of mutation events that are rare and may be preceded by the less visible sub-lineage genetic changes. Nextstrain offers a comprehensive analysis, but is heavily dependent on sequence metadata and dataset pre-filtering. Here we introduce ClusTRace (https://www2.helsinki.fi/en/projects/clustrace), a novel bioinformatic pipeline for Unix/Linux environments that complements the existing toolkits with unsupervised clade or cluster analysis, intuitive visualizations and reporting. ClusTRace can help with surveillance of the current ongoing COVID-19 pandemic and for any upcoming future epidemic or pandemic.

## Implementation

**Fig. 1:**
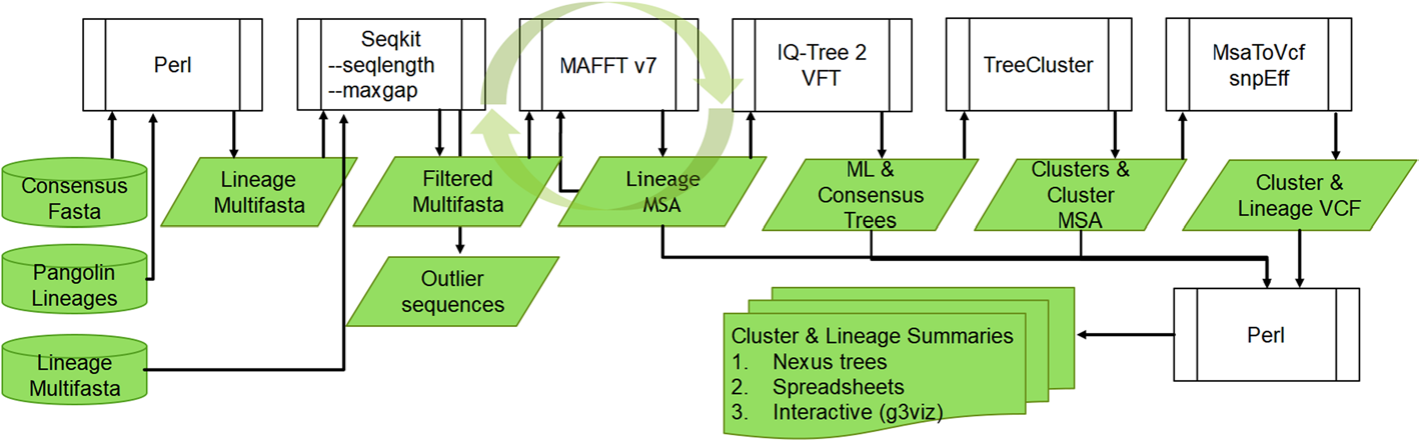
ClusTRace flowchart. VFT, VeryFastTree

ClusTRace is a bioinformatic software package implemented primarily in Perl. ClusTRace supports several tasks that can be executed one by one or combined into pipelines (Fig. 1).

The analysis starts with consensus genomic sequences output by a given sequencing platform (e.g., Illumina). In the first step, ClusTRace assigns genomic sequences to a dynamic Pango lineage classification with Pangolin [20]. Then, ClusTRace collects sequences assigned to different lineages into separate multi-fasta files, so that each multi-fasta contains all sequences assigned to a given Pango lineage. Although we use Pangolin as the default lineage assigner, classification file can be produced with any method preferred by the user (the pipeline will accept any csv-file that conforms to Pangolin output format). All downstream analyses are performed separately for each lineage represented by a multi-fasta file.

Multi-fasta files are then pruned from outliers with SeqKit [26]. By default, we remove all sequences that deviate more than 10% from the median length of the sequence set or that have more than 10% gaps (these parameters can be modified on the command line with *–minlen, –maxlen* and *–maxgap*).

In the next step, filtered sequence sets for each lineage are aligned with MAFFT v7 [27]. Multiple sequence alignments (MSAs) are then trimmed for gaps with trimAl [28]. Trimmed alignments are used to construct phylogenetic trees with IQ-TREE 2 [29]. IQ-TREE 2 supports a wide range of substitution models and will, by default, use ModelFinder to determine the best-fitting model [29]. The user can choose to create bootstrapped consensus trees with IQ-TREE 2 Ultra-Fast Bootstrapping (ClusTRace*–ufboot* option) [30]. For very large sequence sets, the user can choose to run VeryFastTree [31] with GTR model (ClusTRace–*tree vftree* option*)*. By default, ClusTRace will use COVID-19 reference genome (NCBI acc NC_045512.2) as an outgroup sequence to re-root all output phylogenetic trees. There is also an option to specify a separate outgroup sequence for each run.

In the next step, sequence clusters are extracted with TreeCluster [32]. Clusters are extracted with MaxClade-method at several pairwise distance cut-offs. We use two cut-off thresholds that are scaled to the size of the input reference genome (e.g. SARS-CoV-2) and roughly correspond to twenty and thirty mutations between pairs of sequences. MaxClade-method and cut-off thresholds (0.0007 and 0.001) were selected ad hoc based on our previous work with SARS-CoV-2 phylogenies [33]. These values can be easily modified by the user. Next, ClusTRace creates custom nexus trees in which sequences are assigned labels and colours according to the assigned cluster.

ClusTRace can read date annotations from sequence ids and will accept common date formats (e.g. “|YYYY-MM-DD|”). For date annotated sequences ClusTRace will trace the speed of growth for the extracted clusters. This is done by assigning sequences to time periods (calendar months or weeks) and by tracing the number of sequences that are assigned to each cluster and that are dated up to the given time period. For each lineage ClusTRace will print a separate cluster summary file with detailed information on the extracted clusters. These spreadsheet summaries include *clustSeqN*, *clustSeqId* and *clustGR* data sheets*.* The first and second data sheets report the number and ids of sequences in each cluster for each time period, while the third reports cluster size, median and maximal growth rates, and support value for the corresponding sub-phylogeny for each cluster. Separate *clustGR* data sheets are printed for each cluster cut-off threshold (by default twenty and thirty). Median and maximal growth rates are measured based on absolute increment in sequence number assigned to each cluster between consecutive time periods.

In the last step, ClusTRace extracts MSA(s) and runs variant calling for the extracted clusters. Nucleotide mutations are called from these against a reference genome with MsaToVcf [34]. Nucleotide variants are filtered to exclude 100 nucleotides (nt) from the start and the end of the genome (to avoid noise related to sequencing errors commonly seen in terminal regions), as well as any regions that have over 30 nt continuous stretches of below 75% coverage (these are also assumed to represent sequencing errors) using trimAl [28]. We also exclude variants with support below 50%. These filtering options are specified in the pipeline default options and can be modified. Amino acid (aa) variants are called with snpEff [35]. Finally, aa variants in all clusters are parsed and added to the cluster spreadsheet summaries as *clustMutations* and *clustMutationTable* data sheets. The *clustMutations* sheet reports nt and aa mutations for each cluster, reference aa mutations and non-reference aa mutations. Reporting reference and non-reference mutations requires supplying reference mutations in a separate file. For genes of interest non-reference mutations can be reported separately (current version reports mutations for the S-gene). The *clustMutationTable* sheet reports aa mutations for the fastest growing clusters in a binary matrix. The top row lists aa mutations in genomic order with non-reference mutations highlighted in bold.

ClusTRace also supports extracting nt and aa, reference aa and non-reference aa mutations for lineage MSA(s) or for any other collection of MSA(s). Lineage mutations are reported with spreadsheet summary tables similar to the cluster mutation summaries.

ClusTRace also offers an interface to g3viz R library [36]. Using this interface in R, the user can generate interactive mutation plots for both cluster and lineage vcf-files. These interactive plots can be saved in the form of simple html files to complement spreadsheet reports.

## Results

To illustrate the intended use of ClusTRace we analyzed a dataset of SARS-CoV-2 full genome sequences from patient samples collected in Finland from January to June 2021. We started by running ClusTRace Pangolin mapping to obtain 5379 sequences assigned to Alpha and 1051 sequences assigned to Beta variants of concern (VOC) (GISAID accessions are available in Additional file 1: Table S1). We then run ClusTRace multi-fasta construction, outlier filtering, alignment, phylogeny with ultrafast bootstrapping (*–ufboot* option), default clustering and variant calling for these two lineages. As our outgroup sequences we used *EPI_ISL_601443* for the Alpha variant and *EPI_ISL_660190* for the Beta variant. All files output by ClusTRace for this analysis are available in Additional file 2.

**Fig. 2:**
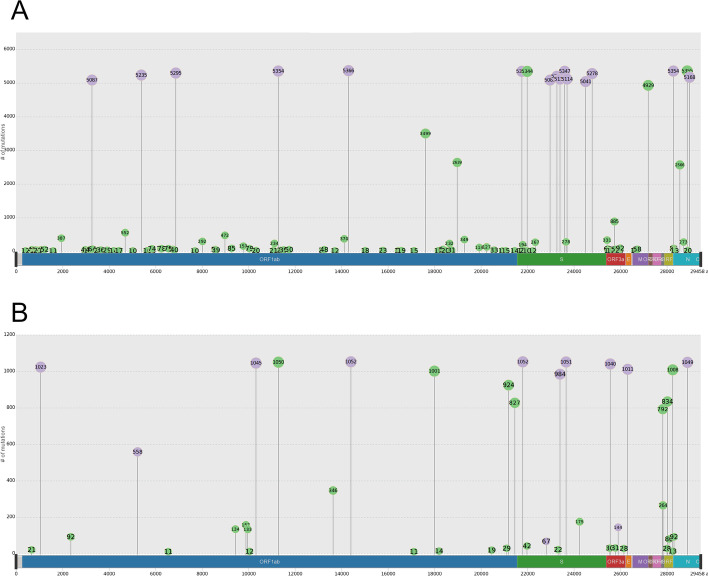
Amino acid mutations for Finnish Alpha (**A**) and Beta (**B**) datasets. Plotting all mutations found in at least ten sequences in Alpha (5379 sequences) and Beta (1051 sequences). Mutations that have been reported as characteristic for a given lineage [37, 38] are plotted in purple, all other mutations are plotted in green. Numbers in cirles indicate the number of sequences with the given mutation. Graphics were created with the ClusTRace interface to g3viz [36]

To get a quick summary on the lineage mutations, we start with g3viz visualisation (Fig. 2, interactive version available in Additional file 2). For Alpha we see that most high frequency aa mutations follow mutations that have been reported as characteristic for this lineage [37] (Fig. 2A). These include T1001I, A1708D, I2230T, 3675_3677del and P4715L in *ORF1ab*, 69_70del, N501Y, A570D, D614G, P681H, T716I, S982A and D1118H in *S*, D3L and S235F in *N*. For Alpha, there are just five aa variants specific for Finnish data with frequency 10% or higher: K5784R and E6272G in *ORF1ab*, N119H in *ORF3a* and G96S and RG203KP in *N*.

For Beta, approximately half of mutations with frequency 10% or higher were not covered by mutations that have been reported as characteristic for this lineage [38] (Fig. 2B). Mutations matching characteristic mutations for Beta were: T265I, K1655N, K3353R and P4715L in *ORF1ab*, D80A, D614G and A701V in *S*, Q57H and S171L in *ORF3a*, P71L in *E*, T205I in *N*, while the non-characteristic aa mutations with at least 10% frequency were: T3058I, A3209V, A3235S, D4459A, T5912I and A6976V in *ORF1ab*, T19I and I896L in *S*, M24V, I26V and I27V in *ORF7b*, K44R and I121L in *ORF8*. Note that Beta has non-characteristic mutations in Spike protein, which may potentially affect their receptor binding: T19I in 789 (75%) and I896L in 175 (16.7%) sequences.

**Fig. 3:**
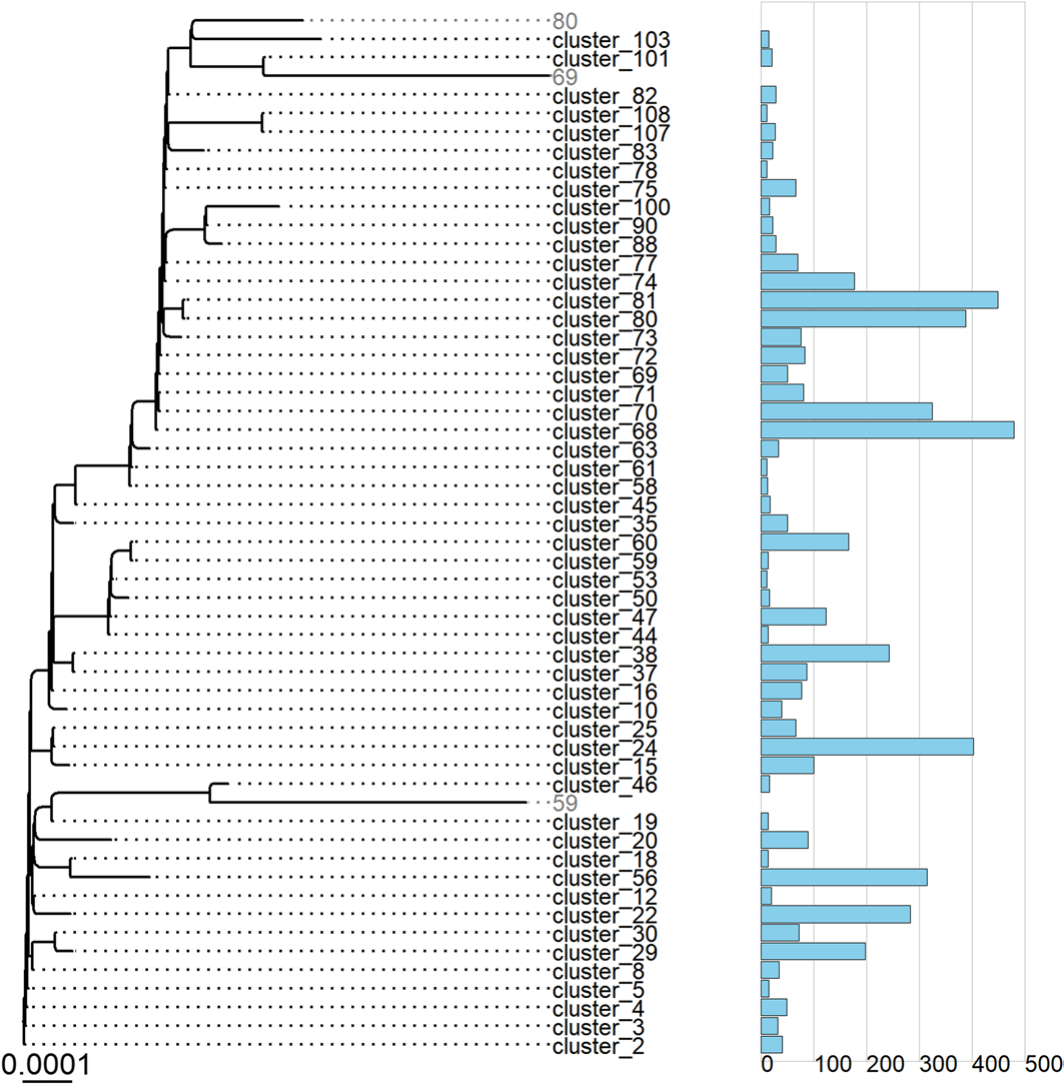
Consensus tree for Finnish Alpha dataset with clusters collapsed. Bar plots on the right indicate the number of sequences in each cluster. For clarity, clusters with less than ten sequences and singletons were removed. Inner nodes with no large cluster descendants are plotted in grey

**Fig. 4:**
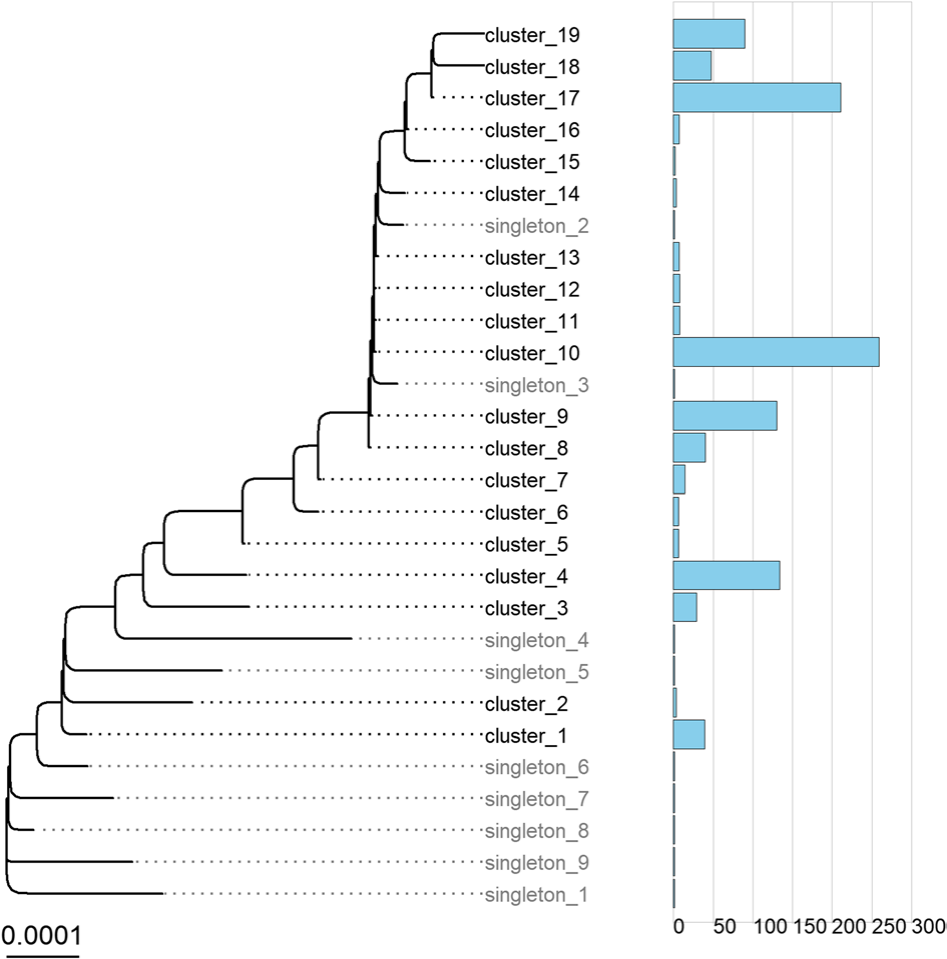
Consensus tree for Finnish Beta dataset with clusters collapsed. Bar plots on the right indicate the number of sequences in each cluster. For clarity, clusters with less than ten sequences were removed

Cluster analysis with TreeCluster [32] yielded 108 clusters for Alpha and nineteen clusters for Beta (Figs. 3 and 4, full consensus trees with clusters highlighted are available in files B.1.1.7.con.tree.mr = 30.nex and B.1.351.con.tree.mr = 30.nex in Additional file 2). We used the MaxClade method with a cut-off set to 0.001. Here we take a closer look at the ten clusters for Alpha and Beta that had the highest per month growth rate peaks over the analysed time period.

**Fig. 5:**
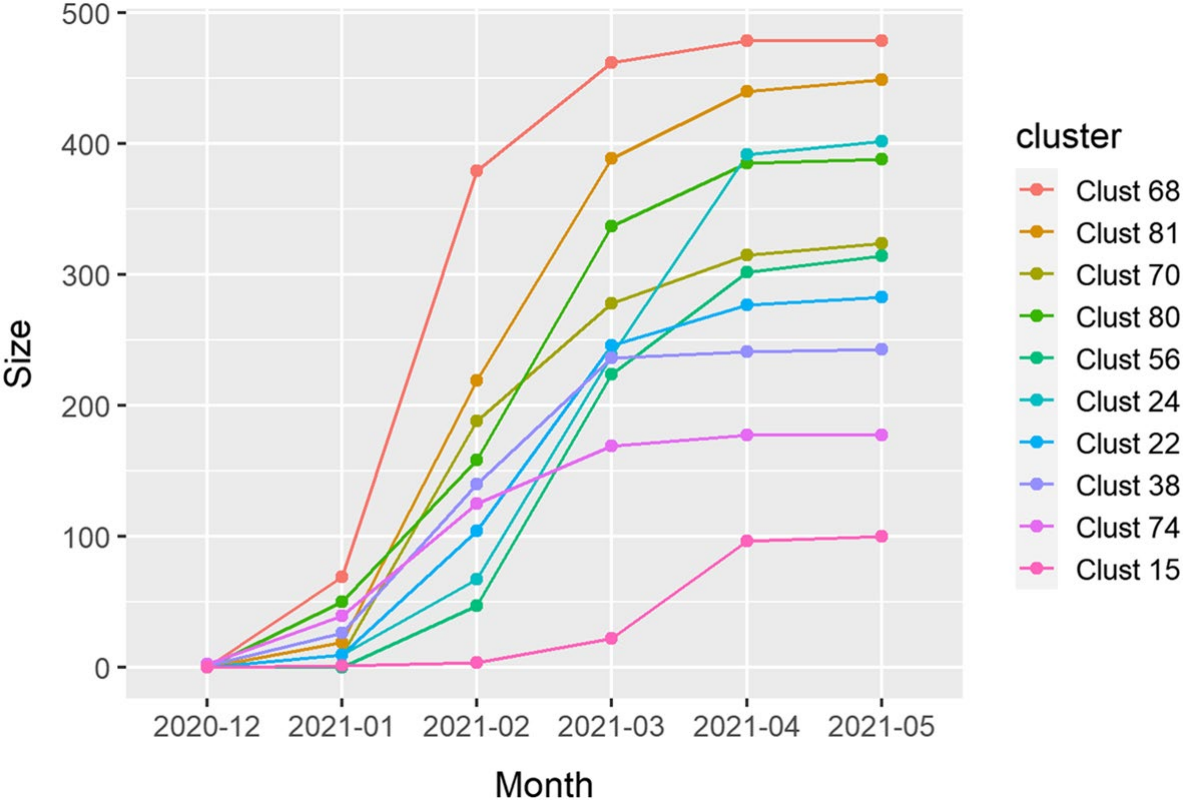
Growth rates for the ten fastest growing clusters in Alpha (B.1.1.7)

We start by discussing Alpha clusters. The ten fastest growing clusters covered 3,146 (58.5%) of all Alpha sequences. Cluster size varied in these ten clusters between 100 (1.9%) and 479 (8.9%) sequences (Fig. 5). Maximal growth rates ranged between 74 and 310 sequences per month and peak growth was during February and March. Number of non-characteristic aa mutations introduced in these clusters ranged from one to six. Solitary non-characteristic mutations in S-gene were found in clusters 56 (D80Y), 38 (D287G) and 22 (A701V) (Table 1).

**Table 1:** Mutations in Alpha (B.1.1.7) clusters

Cluster	S										
	**L18F**	**T19I**	D80A	D215G	241_243del	K417N	E484K	N501Y	D614G	A701V	**I896L**
*GISAID B.1.351	0	0	1	1	1	1	1	1	1	1	0
Clust 10	0	1	1	1	1	0	1	1	1	1	0
Clust 17	0	1	1	1	1	0	1	1	1	1	0
Clust 19	0	1	1	0	0	0	1	1	1	1	0
Clust 9	0	1	1	1	1	0	1	1	1	1	1
Clust 4	0	0	1	1	1	0	1	1	1	1	0
Clust 1	1	0	1	1	1	0	1	1	1	1	0
Clust 8	0	1	1	1	1	0	1	1	1	1	0
Clust 18	0	1	1	1	1	0	0	0	1	1	0
Clust 3	0	0	1	1	1	0	1	1	1	1	0
Clust 7	0	0	1	1	1	0	1	1	1	1	0

Cluster	S										
	**L18F**	**T19I**	D80A	D215G	241_243del	K417N	E484K	N501Y	D614G	A701V	**I896L**
*GISAID B.1.351	0	0	1	1	1	1	1	1	1	1	0
Clust 10	0	1	1	1	1	0	1	1	1	1	0
Clust 17	0	1	1	1	1	0	1	1	1	1	0
Clust 19	0	1	1	0	0	0	1	1	1	1	0
Clust 9	0	1	1	1	1	0	1	1	1	1	1
Clust 4	0	0	1	1	1	0	1	1	1	1	0
Clust 1	1	0	1	1	1	0	1	1	1	1	0
Clust 8	0	1	1	1	1	0	1	1	1	1	0
Clust 18	0	1	1	1	1	0	0	0	1	1	0
Clust 3	0	0	1	1	1	0	1	1	1	1	0
Clust 7	0	0	1	1	1	0	1	1	1	1	0

Cluster	ORF3a					E	ORF7b			ORF8			N	
	**L52F**	Q57H	**W131L**	S171L	G254*	P71L	**M24V**	**I26V**	**I27V**	**P38S**	**K44R**	**I121L**	**A12T**	T205I
*GISAID B.1.351	0	1	0	1	0	1	0	0	0	0	0	0	0	1
Clust 10	0	1	0	0	0	1	1	1	1	0	1	1	0	1
Clust 17	0	1	0	0	0	1	1	0	0	0	1	1	0	1
Clust 19	0	1	0	0	0	1	1	0	0	0	1	1	1	1
Clust 9	0	1	0	0	0	1	1	0	0	0	1	1	0	1
Clust 4	0	1	0	0	0	1	0	0	0	0	0	1	0	1
Clust 1	0	1	1	0	1	1	0	0	0	0	0	0	0	1
Clust 8	0	1	0	0	0	1	1	0	0	0	1	1	0	1
Clust 18	0	1	0	0	0	1	1	1	1	0	1	1	0	1
Clust 3	1	1	0	0	0	1	0	0	0	1	0	1	0	1
Clust 7	0	1	0	0	0	1	0	0	0	0	1	1	0	1

Here, aa mutations with frequencies exceeding 50% are listed in genomic order

*The first row depicts mutations characteristic for B.1.1.7 according to the lineage report [37]

**Fig. 6:**
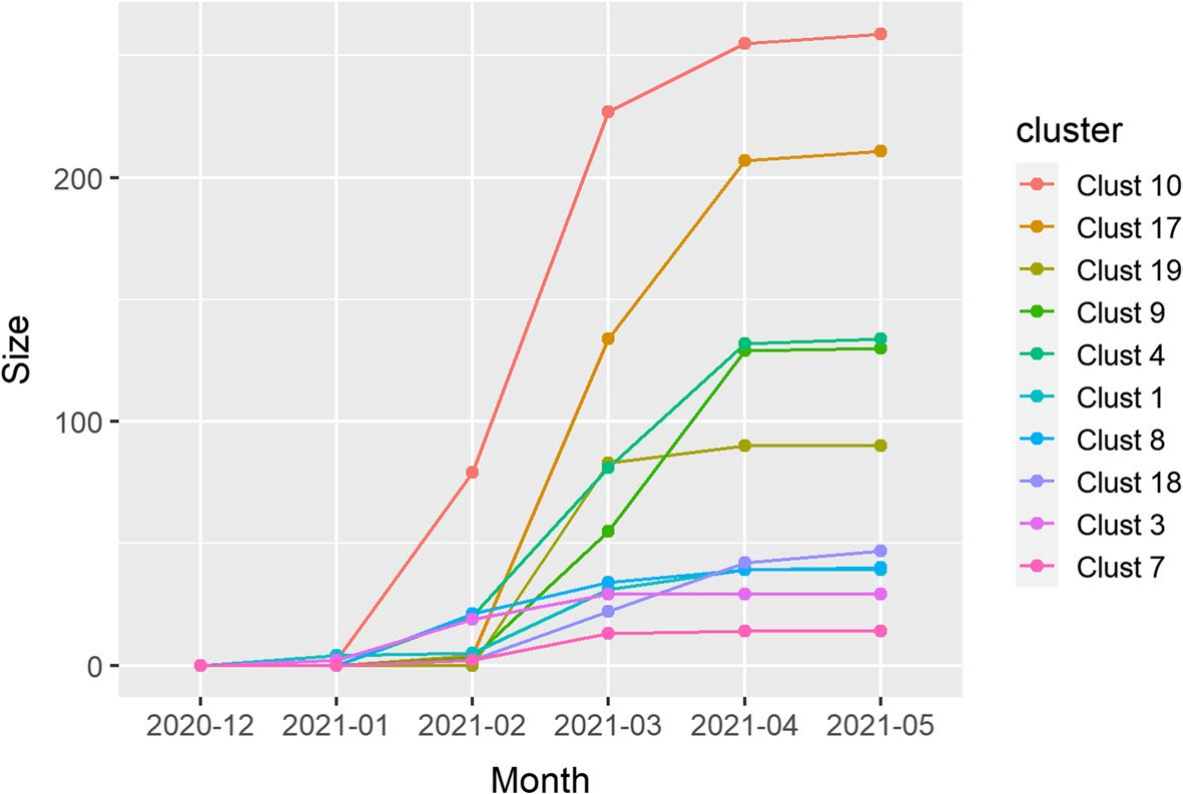
Growth rates for the ten fastest growing clusters in Beta (B.1.351)

The ten fastest growing clusters covered 979 (94.5%) of Beta sequences. Cluster size was between fourteen (1.3%) and 259 (24.6%) sequences (Fig. 6). Maximal growth rates ranged between 11 and 148 sequences per month and maximal growth was during February (clusters 3 and 8), March (clusters 1, 4, 7, 10, 17, 18 and 19) and April (cluster 9). Number of non-characteristic aa mutations introduced in these clusters ranged from three to eight. Several clusters had non-characteristic mutations in S-gene: L18F (cluster 1), T19I (clusters 8–10, 17 and 19) and I896L (cluster 9) (Table 2).

**Table 2:** Mutations in Beta clusters

Cluster	S										
	**L18F**	**T19I**	D80A	D215G	241_243del	K417N	E484K	N501Y	D614G	A701V	**I896L**
*GISAID B.1.351	0	0	1	1	1	1	1	1	1	1	0
Clust 10	0	1	1	1	1	0	1	1	1	1	0
Clust 17	0	1	1	1	1	0	1	1	1	1	0
Clust 19	0	1	1	0	0	0	1	1	1	1	0
Clust 9	0	1	1	1	1	0	1	1	1	1	1
Clust 4	0	0	1	1	1	0	1	1	1	1	0
Clust 1	1	0	1	1	1	0	1	1	1	1	0
Clust 8	0	1	1	1	1	0	1	1	1	1	0
Clust 18	0	1	1	1	1	0	0	0	1	1	0
Clust 3	0	0	1	1	1	0	1	1	1	1	0
Clust 7	0	0	1	1	1	0	1	1	1	1	0

Cluster	S										
	**L18F**	**T19I**	D80A	D215G	241_243del	K417N	E484K	N501Y	D614G	A701V	**I896L**
*GISAID B.1.351	0	0	1	1	1	1	1	1	1	1	0
Clust 10	0	1	1	1	1	0	1	1	1	1	0
Clust 17	0	1	1	1	1	0	1	1	1	1	0
Clust 19	0	1	1	0	0	0	1	1	1	1	0
Clust 9	0	1	1	1	1	0	1	1	1	1	1
Clust 4	0	0	1	1	1	0	1	1	1	1	0
Clust 1	1	0	1	1	1	0	1	1	1	1	0
Clust 8	0	1	1	1	1	0	1	1	1	1	0
Clust 18	0	1	1	1	1	0	0	0	1	1	0
Clust 3	0	0	1	1	1	0	1	1	1	1	0
Clust 7	0	0	1	1	1	0	1	1	1	1	0

Cluster	ORF3a					E	ORF7b			ORF8			N	
	**L52F**	Q57H	**W131L**	S171L	G254*	P71L	**M24V**	**I26V**	**I27V**	**P38S**	**K44R**	**I121L**	**A12T**	T205I
*GISAID B.1.351	0	1	0	1	0	1	0	0	0	0	0	0	0	1
Clust 10	0	1	0	0	0	1	1	1	1	0	1	1	0	1
Clust 17	0	1	0	0	0	1	1	0	0	0	1	1	0	1
Clust 19	0	1	0	0	0	1	1	0	0	0	1	1	1	1
Clust 9	0	1	0	0	0	1	1	0	0	0	1	1	0	1
Clust 4	0	1	0	0	0	1	0	0	0	0	0	1	0	1
Clust 1	0	1	1	0	1	1	0	0	0	0	0	0	0	1
Clust 8	0	1	0	0	0	1	1	0	0	0	1	1	0	1
Clust 18	0	1	0	0	0	1	1	1	1	0	1	1	0	1
Clust 3	1	1	0	0	0	1	0	0	0	1	0	1	0	1
Clust 7	0	1	0	0	0	1	0	0	0	0	1	1	0	1

Annotation as in Table 1

### Benchmarking time and memory efficiency

We benchmarked ClusTRace performance on two datasets with default settings on a Red Hat Enterprise Linux Server 7.9 on a single node with 32 × 2.1 GHz cores. The first dataset included 6,430 SARS-CoV-2 genomic sequences from patient samples collected in Finland from January to June 2021 (GISAID accession ids are available in Additional file 1: Table S1). This run completed in 48 h and 6 min and consumed 83.26 GB of memory. The second dataset included 3,568 genomic sequences for Delta variant sequenced from Finnish patient samples later the same year (GISAID accession ids are available in Additional file 3: Table S2). This run completed in 14 h and 16 min and consumed 75.44 GB of memory. Most time was spent within IQ-Tree calls. We see that execution time does seem to scale nonlinearly with dataset size but is kept within acceptable limits for moderately large datasets. The required memory usage for these datasets was well below available limits.

## Discussion

The years 2020 and 2021 could arguably be referred to as a turning point in the history of global health. The COVID-19 pandemic has demonstrated that emerging pathogens can cause havoc in our globalised world. On the other hand, the pandemic has also accelerated the development of better sequencing technologies, bioinformatic tools, diagnostic tests, vaccines and many other fields. The ongoing pandemic has emphasised the need for fast, scalable and, ideally pipelined, analysis of viral genomic sequences. For health authorities, it is important to be able to streamline processing large amounts of genomic sequence data into various summaries and reports that can help to make rational decisions concerning e.g. restrictions, non-pharmaceutical interventions and border control measures to minimize further spread of SARS-CoV-2. Researchers also struggle with the continuous inflow of SARS-CoV-2 sequences that need to be organized into lineages, alignments and phylogenetic trees in order to make sense of the constantly evolving pandemic.

Here, we have presented ClusTRace, a novel bioinformatic pipeline for fast and scalable analysis of large collections of SARS-CoV-2 sequences. ClusTRace supports many types of relevant analyses. These include assigning sequences to lineages, collecting sequences by lineage, filtering outliers, creating multiple sequence alignments, creating phylogenetic trees, extracting phylogeny-based sequence clusters, estimating cluster growth rates, calling nt and aa variants for both lineages and clusters, as well generating a number of table-based and interactive reports. Although most of these steps can be performed separately with designated bioinformatic tools, pipelining with a high-level interface helps to cut down on the learning and operating costs of complex bioinformatic analysis. Several authors have commented on the developer-user gap between bioinformatics and other fields in biology and biomedical research [10]. In this context, high-level pipelines that are tailored to the need of virus research are an important way to bridge this gap.

Popular pipelines for tracking viral outbreak phylodynamics include Augur, Auspice, Nextstrain, Nextclade and Pangolin [20–22, 39]. Here, we reflect on key similarities and differences of ClusTRace to these toolkits. Pangolin and Nextclade are primarily concerned with classifying viral genomes into lineages or clades, while ClusTRace is designed to track mutations within lineages. Nextclade also offers mutation calling for large clades, which is similar to ClusTRace mutation calling for lineages. Nextstrain is an integration of several toolkits, including Augur for analysing sequence and phylogeographical data, and Auspice for visualising results. Like ClusTRace, Augur offers functionalities for filtering, aligning, phylogenetic reconstruction, re-rooting and refinement of the obtained phylogenies, and offers functionalities to estimate mutation frequencies. Unlike ClusTRace, Augur also infers sequences and ancestral traits for the ancestral tree nodes. Auspice is designed to visualise phylogenetic and phylogeographic data output by Augur in an interactive webpage format. In ClusTRace, we provide different visualizations, namely spreadsheet summaries and interactive g3viz plots for high growth-rate and/or mutation-rate clades. Unlike Nextstrain/Auspice visualizations, ClusTRace focuses directly on parts of the phylogeny that are picked out by the unsupervised cluster analysis and provides no details on the likely origin of the mutations in the tree. However, this approach has its advantages, such as simplicity and speed; unlike Nextstrain/Augur, ClusTRace has no need for down sampling the sequence sets. ClusTRace analysis is also largely unsupervised, i.e. clades are selected and examined for mutations and growth-peaks automatically, in effect filtering clades with alarming features that can then be checked manually more in detail.

In this work, we illustrated the intended scenario for ClusTRace usage on Finnish Alpha and Beta variants of concern. Presented approach can be described as an unsupervised phylogeny-based cluster analysis and variant calling. ClusTRace uses automated unsupervised clustering coupled with cluster growth rate analysis and variant calling to scan through the phylogeny. Clusters that display elevated growth rates, elevated non-reference mutation content or mutations in genomic regions that are of accentuated concern, such as the S-gene, can then be flagged for downstream analysis. In this paper we focus on describing the method and do not attempt to link identified cluster to epidemiologic seeding events. However, in our other work on monitoring SARS-CoV-2 spread in Finland we have appleid identical clustering with some success. For example, in [33] we monitored clusters for Alpha and Beta lineages and in that work clustering suggested that these lineages have spread to Finland via multiple seeding events. In our analysis of Finnish Omicron sequences we were able to identify a single large cluster that most likely corresponded to a super-spreading event (n = 236, which is 27.1% of all Finnish cases) as well as numerous smaller clusters that indicate multiple seeding points [40].

The current SARS-CoV-2 pandemic might endure to the foreseeable future, and new viral variants will likely continue to emerge. Therefore, the global response must continue to adapt and improve to mitigate the costs of the pandemic. The progress made since the start of the pandemic in early 2020 with the global implementation of full genome sequencing can be consolidated by developing efficient and scalable bioinformatic tools that are specifically tailored for genomic surveillance of viral pathogens. These tools must deliver fast, scalable and, ideally, unsupervised analysis and reporting on the pandemic events of concern. Our pipeline, ClusTRace, adds to the available toolbox the option for fast, scalable and unsupervised screening and reporting of the within or local lineage events of concern, such as elevated growth and mutation rates. ClusTRace can also be adapted for the surveillance of viral pathogens other than the SARS-CoV-2, which may prove useful in future epidemic emergencies.

### Availability and requirements

Project name: ClusTRace.

Project home page: https://bitbucket.org/plyusnin/clustrace/src/master/;

https://www2.helsinki.fi/en/projects/clustrace/

Operating system: Linux.

Programming language: Perl.

License: GNU GPL.

Other requirements: listed on project home page.

## Supplementary Information

**Additional file 1**: Table 1. Alfa and Beta sequences from Finland. GISAID accession ids for Alfa and Beta variants of concern genomic sequences from Finland.

**Additional file 2**: ClusTRace results for Alfa and Beta sequences. Includes all files output by ClusTRace.

**Additional file 3**: Table 2. Delta sequences from Finland. GISAID accession ids for Delta variant of concern genomic sequences from Finland.

## Data Availability

GISAID accession ids for both datasets referred in the main text are listed in Additional files 1 and 3 Tables S1 and S2. These sequences are publicly available in GISAID database (https://www.gisaid.org/).

## Abbreviations

COVID-19: Coronavirus disease 2019
MSA: Multiple sequence alignment
VOC: Variant of Concern
VOI: Variant of Interest
SARS-CoV-2: Severe acute respiratory syndrome coronavirus 2

## References

[1] Dixon MG, Schafer IJ (2014). Centers for disease control and prevention (CDC). Ebola viral disease outbreak–West Africa, 2014. MMWR Morb Mortal Wkly Rep.

[2] Kindhauser MK, Allen T, Frank V, Santhana RS, Dye C (2016). Zika: the origin and spread of a mosquito-borne virus. Bull World Health Organ.

[3] Woolhouse MEJ, Gowtage-Sequeria S (2005). Host range and emerging and reemerging pathogens. Emerg Infect Dis J - CDC.

[4] Schmeller DS, Courchamp F, Killeen G (2020). Biodiversity loss, emerging pathogens and human health risks. Biodivers Conserv.

[5] Jones KE, Patel NG, Levy MA, Storeygard A, Balk D, Gittleman JL (2008). Global trends in emerging infectious diseases. Nature.

[6] Morens DM, Fauci AS. Emerging pandemic diseases: how we got to COVID-19. Cell. 2020. 10.1016/j.cell.2020.10.022 PMC7598893 33125895

[7] Fleischmann Jr WR. Viral genetics. In: Medical Microbiology. 4th edition. University of Texas Medical Branch at Galveston; 1996. p. Chapter 43.

[8] Quick J, Loman NJ, Duraffour S, Simpson JT, Severi E, Cowley L (2016). Real-time, portable genome sequencing for Ebola surveillance. Nature.

[9] Oude Munnink BB, Worp N, Nieuwenhuijse DF, Sikkema RS, Haagmans B, Fouchier RAM (2021). The next phase of SARS-CoV-2 surveillance: real-time molecular epidemiology. Nat Med.

[10] Mangul S, Martin LS, Hill BL, Lam AK-M, Distler MG, Zelikovsky A (2019). Systematic benchmarking of omics computational tools. Nat Commun.

[11] Wu F, Zhao S, Yu B, Chen Y-M, Wang W, Song Z-G (2020). A new coronavirus associated with human respiratory disease in China. Nature.

[12] Wise J (2020). Covid-19: new coronavirus variant is identified in UK. BMJ.

[13] Tegally H, Wilkinson E, Giovanetti M, Iranzadeh A, Fonseca V, Giandhari J (2020). Emergence and rapid spread of a new severe acute respiratory syndrome-related coronavirus 2 (SARS-CoV-2) lineage with multiple spike mutations in South Africa. medRxiv.

[14] Faria NR, Claro IM, Candido D, Franco LM, Andrade PS, Coletti TM (2021). Genomic characterisation of an emergent SARS-CoV-2 lineage in Manaus: preliminary findings. Virological.

[15] Kirola L (2021). Genetic emergence of B.1617.2 in COVID-19. New Microb New Infect.

[16] Latif AA, Mullen JL, Manar A, Tsueng G, Cano M, Emily H, et al. B.1.1.529 Lineage Report (available at https://outbreak.info/situation-reports?pango=B.1.1.529). Accessed 30 November 2021. 2021.

[17] Campbell F, Archer B, Laurenson-Schafer H, Jinnai Y, Konings F, Batra N (2021). Increased transmissibility and global spread of SARS-CoV-2 variants of concern as at June 2021. Eurosurveillance.

[18] Virtanen J, Uusitalo R, Korhonen EM, Aaltonen K, Smura T, Kuivanen S (2021). Kinetics of neutralizing antibodies of COVID-19 patients tested using clinical D614G, B.1.1.7, and B 1.351 isolates in microneutralization assays. Viruses.

[19] Jalkanen P, Kolehmainen P, Häkkinen HK, Huttunen M, Tähtinen PA, Lundberg R (2021). COVID-19 mRNA vaccine induced antibody responses against three SARS-CoV-2 variants. Nat Commun.

[20] O’Toole Á, Scher E, Underwood A, Jackson B, Hill V, McCrone JT (2021). Assignment of epidemiological lineages in an emerging pandemic using the pangolin tool. Virus Evol.

[21] Hadfield J, Megill C, Bell SM, Huddleston J, Potter B, Callender C (2018). Nextstrain: real-time tracking of pathogen evolution. Bioinformatics.

[22] Aksamentov I, Roemer C, Hodcroft EB, Neher RA (2021). Nextclade: clade assignment, mutation calling and quality control for viral genomes. J Open Sour Softw.

[23] Zwagemaker F, Schmitz D, Nooij S, kroonma, Laros JFJ. DennisSchmitz/Jovian: 1.2.07. Zenodo; 2021.

[24] Nguyen PTT, Plyusnin I, Sironen T, Vapalahti O, Kant R, Smura T (2021). HAVoC, a bioinformatic pipeline for reference-based consensus assembly and lineage assignment for SARS-CoV-2 sequences. BMC Bioinform.

[25] Plyusnin I, Kant R, Jääskeläinen AJ, Sironen T, Holm L, Vapalahti O (2020). Novel NGS pipeline for virus discovery from a wide spectrum of hosts and sample types. Virus Evolut.

[26] Shen W, Le S, Li Y, Hu F (2016). SeqKit: a cross-platform and ultrafast toolkit for FASTA/Q file manipulation. PLoS ONE.

[27] Katoh K, Standley DM (2013). MAFFT multiple sequence alignment software version 7: improvements in performance and usability. Mol Biol Evol.

[28] Capella-Gutiérrez S, Silla-Martínez JM, Gabaldón T (2009). trimAl: a tool for automated alignment trimming in large-scale phylogenetic analyses. Bioinformatics.

[29] Minh BQ, Schmidt HA, Chernomor O, Schrempf D, Woodhams MD, von Haeseler A (2020). IQ-TREE 2: new models and efficient methods for phylogenetic inference in the genomic era. Mol Biol Evol.

[30] Hoang DT, Chernomor O, Von Haeseler A, Minh BQ, Vinh LS (2018). UFBoot2: improving the ultrafast bootstrap approximation. Mol Biol Evol.

[31] Piñeiro C, Abuín JM, Pichel JC (2020). Very Fast Tree: speeding up the estimation of phylogenies for large alignments through parallelization and vectorization strategies. Bioinformatics.

[32] Balaban M, Moshiri N, Mai U, Jia X, Mirarab S (2019). TreeCluster: clustering biological sequences using phylogenetic trees. PLoS ONE.

[33] Kant R, Nguyen PT, Blomqvist S, Erdin M, Alburkat H, Suvanto M (2021). Incidence trends for SARS-CoV-2 Alpha and Beta variants, Finland, spring 2021. Emerg Infect Dis.

[34] Page AJ, Taylor B, Delaney AJ, Soares J, Seemann T, Keane JA (2016). SNP-sites: rapid efficient extraction of SNPs from multi-FASTA alignments. Microb Genom.

[35] Cingolani P, Platts A, Wang LL, Coon M, Nguyen T, Wang L (2012). A program for annotating and predicting the effects of single nucleotide polymorphisms, SnpEff: SNPs in the genome of Drosophila melanogaster strain w1118; iso-2; iso-3. Fly.

[36] Guo X, Zhang B, Zeng W, Zhao S, Ge D (2020). G3viz: an R package to interactively visualize genetic mutation data using a lollipop-diagram. Bioinformatics.

[37] Latif AA, Mullen JL, Manar A, Tsueng G, Cano M, Emily H, et al. B.1.1.7 Lineage Report. outbreak.info, (https://outbreak.info/situation-reports?pango=B.1.1.7). Accessed 28 September 2021. 2021.

[38] Latif AA, Mullen JL, Manar A, Tsueng G, Cano M, Emily H, et al. B.1.351 Lineage Report. outbreak.info, (https://outbreak.info/situation-reports?pango=B.1.351). Accessed 28 September 2021. 2021.

[39] Huddleston J, Hadfield J, Sibley TR, Lee J, Fay K, Ilcisin M (2021). Augur: a bioinformatics toolkit for phylogenetic analyses of human pathogens. J Open Sour Softw.

[40] Vauhkonen H, Truong P, Kant R, Plyusnin I, Erdin M, Kurkela S, et al. Introduction and rapid spread of SARS-CoV-2 Omicron variant and the dynamics of its sub-lineages BA.1 and BA.1.1, December 2021, Finland. 2022. 10.3201/eid2806.220515 PMC9155872 35378057

