# Supplementary material for: ClusTRace, a bioinformatic pipeline for analyzing clusters in virus phylogenies
Source: BMC Bioinformatics. 2022 May 28;23:196. doi: 10.1186/s12859-022-04709-8 (PMC9143711; doi:10.1186/s12859-022-04709-8)
Supplement: Supplementary file 2 — Additional file 2: ClusTRace results for Alfa and Beta sequences. Includes all files output by ClusTRace. [file 12859_2022_4709_MOESM2_ESM.zip › FIN-SC2-acc210624/B.1.351.mutations.html]

g3Lollipop
